# SalHUD—A Graphical Interface to Public Health Data in Puerto Rico †

**DOI:** 10.3390/ijerph13010018

**Published:** 2015-12-22

**Authors:** Humberto G. Ortiz-Zuazaga, Roberto Arce-Corretjer, Juan M. Solá-Sloan, José G. Conde

**Affiliations:** 1 Department of Computer Science, University of Puerto Rico, Río Piedras Campus, San Juan 00936, Puerto Rico; racorretjer@gmail.com; 2 Department of Computer Science, University of Puerto Rico, Bayamón Campus, Bayamón 00959, Puerto Rico; juan.sola@upr.edu; 3 School of Medicine, University of Puerto Rico, Medical Sciences Campus, San Juan 00936, Puerto Rico; jose.conde1@upr.edu

**Keywords:** health data, visualization, Javascript

## Abstract

Purpose: This paper describes SalHUD, a prototype web-based application for visualizing health data from Puerto Rico. Our initial focus was to provide interactive maps displaying years of potential life lost (YPLL). Methods: The public-use mortality file for year 2008 was downloaded from the Puerto Rico Institute of Statistics website. Data was processed with R, Python and EpiInfo to calculate years of potential life lost for the leading causes of death on each of the 78 municipalities in the island. Death records were classified according to ICD-10 codes. YPLL for each municipality was integrated into AtlasPR, a D3 Javascript map library. Additional Javascript, HTML and CSS programing was required to display maps as a web-based interface. Results: YPLL for all municipalities are displayed on a map of Puerto Rico for each of the ten leading causes of death and for all causes combined, so users may dynamically explore the impact of premature mortality. Discussion: This work is the first step in providing the general public in Puerto Rico with user-friendly, interactive, visual access to public health data that is usually published in numerical, text-based media.

## 1. Introduction

Under the Health Data Initiative, the U.S. Department of Health and Human Services releases large amounts of health data for public use. The mission of the initiative is “to improve health, health care, and the delivery of human services by harnessing the power of data and fostering a culture of innovative uses of data in public and private sector institutions, communities, research groups and policy making arenas” [1]. Software developers use data to create applications that make health information increasingly useful for individuals, communities, service providers and policy-makers [1].

This paper describes SalHUD, a prototype web-based application for visualizing health data from Puerto Rico. Our initial focus was to provide interactive maps displaying years of potential life lost (YPLL) from leading causes of death by municipality. YPLL is a measure of premature mortality in the population under study. YPLL is not reported by the Department of Health in Puerto Rico as part of its annual statistics reports. The most recent publication about YPLL in Puerto Rico dates from 1992 [2]. In order to bring attention to this issue, we decided to choose YPLL in Puerto Rico as the first health indicator to be displayed in SalHUD.

## 2. Methods

**Figure 1:**
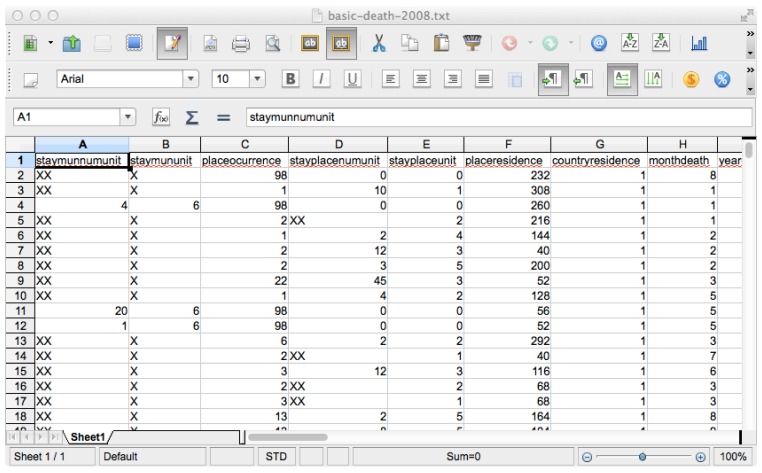
A portion of the Puerto Rico Basic Mortality file for year 2008. The full file contains 29,100 records.

The public-use Puerto Rico Basic Mortality file for year 2008, containing unidentified data from death certificates of 29,100 deaths, was downloaded from the Puerto Rico Institute of Statistics website [3]. Mortality files from Puerto Rico are generated by the Puerto Rico Department of Health following standards of the National Vital Statistics System, National Center of Health Statistics, Centers of Disease Control and Prevention. Deaths are recorded according to underlying cause of death codes from the International Classification of Diseases (ICD-10) [4]. Data are published as comma-separated value (.csv) files, one line per death record, with age at death coded into two columns (namely, type of age units such as minutes, hours, days, weeks, months or years; and number of units). Municipality of residence at time of death is coded with a three-digit number, and underlying cause of death is coded into four columns containing ICD-10 codes. See Figure 1 for a portion of one of the files.

Data was processed with Python [5] to extract the municipality of residence, the age in years at death, and categorize the cause of death into one of the 10 leading causes of death in Puerto Rico for each record. The python program then computes YPLL before the age 75 for each record, and prepares a table with the total YPLL for each leading cause of death, and for all causes combined, per municipality. YPLL was calculated by subtracting the age at death from 75 [6]. Starting in 1996, the National Center for Health Statistics (NCHS) has been presenting YPLL for persons under age 75 because the average life expectancy in the United States is over 75 years [7]. We decided to do the same in order to provide numbers that are comparable to those reported by NCHS. In addition, life expectancy in Puerto Rico for 2008 was 78 years [8].

We constructed an R [9] prototype application to construct a choropleth, a map of Puerto Rico shaded by combined YPLL in each municipality, as a proof of concept. This choropleth was static, and displayed only the total YPLL for each municipality, not the YPLL for a particular cause of death. EpiInfo [10] was used to validate the python program and check the YPLL values we had calculated. The final interactive maps were developed with AtlasPR, a D3 Javascript map library [11]. Additional Javascript, Hyper Text Markup Language (HTML) and Cascading Style Sheet (CSS) programming was required to display maps as a web-based interface where users can select YPLL from all causes combined or one of the 10 leading causes of death, and have the map update dynamically.

## 3. Results and Discussion

**Figure 2:**
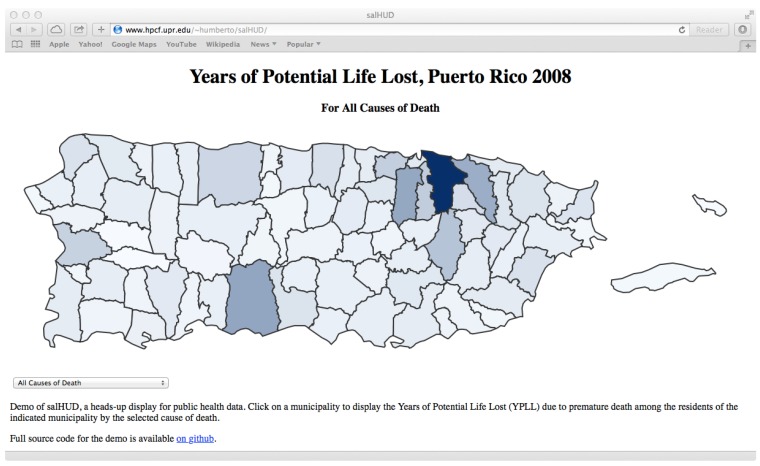
Screenshot of the SalHUD demo application shaded by all causes years of potential life lost (YPLL) in each municipality in Puerto Rico.

YPLL for all municipalities are displayed on a map of Puerto Rico for each of the ten leading causes of death and for all causes combined, so users may dynamically explore the impact of premature mortality (see Figure 2). A drop-down menu provides a list of causes of death to select what YPLL map to view. Map segments representing municipal territories are shaded in a gradient based on a linear interpolation between minimum and maximum values of the distribution of YPLL among municipalities.

**Figure 3:**
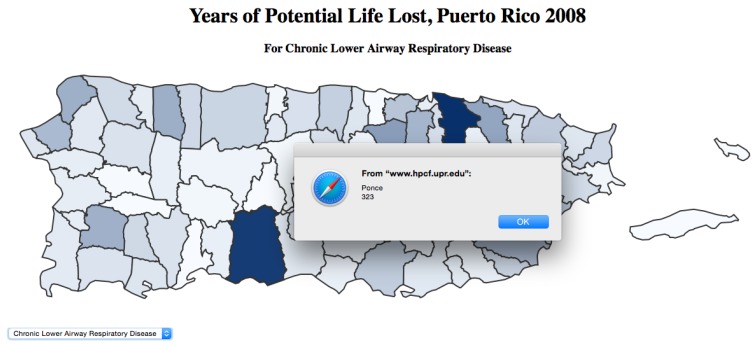
Screenshot of the SalHUD demo application showing the total YPLL for Chronic Lower Respiratory Airway Disease in the municipality of Ponce in 2008.

In addition, the actual number of YPLL for each municipality is integrated to each area, so that clicking on any segment of the map activates a pop-up window displaying the YPLL for the municipality. Figure 3 shows the pop-up dialog box.

A video of the application is available at https://www.youtube.com/watch?v=YIXvXLQTZFw. A working prototype is available at: http://www.hpcf.upr.edu/ humberto/salHUD/. The full source code for the application is available as well [12].

The resulting application displays a summary of the impact of premature death by the leading causes of death in Puerto Rico per municipality. This display is much more accessible to non-technical users than the original data files (See Figure 1). In addition, presenting the data in a graphical format is a major improvement over the usual tabular layout commonly used in vital statistics reports, minimizing information overload and optimizing extraction of information from data.

One key component of the Health Data Initiative is to foster a culture of innovative uses of data at the community level [1]. This can only be achieved by presenting data in a manner that is easy to understand and that engages citizens from all sectors and ranges of society in the analysis of data for the common good. SalHUD is a step in that direction.

## 4. Conclusions

SalHUD displays YPLL distribution among municipalities in Puerto Rico in an interactive, graphical environment, stimulating exploration and engagement by the user. This is the first step in providing the general public in Puerto Rico with user-friendly, dynamic access to public health data that is usually published in numerical, text-based media. A similar approach can be used to visualize other types of health data and to visualize health indicators in other communities.

## References

[1] Health Data—HHS Idea Lab. http://www.hhs.gov/idealab/what-we-do/health-data/.

[2] Ramirez de Arellano A.B. (1992). The death divide: Differentials in premature mortality by gender in Puerto Rico. Bol. Asoc. Med. P. R..

[3] Instituto de Estadisticas de Puerto Rico (2008). Basic Mortality. http://www.estadisticas.gobierno.pr/iepr/Estadisticas/Basesdedatos/Salud.aspx#CDC_mort_ba.

[4] International Statistical Classification of Diseases and Related Health Problems 10th Revision (ICD-10). http://apps.who.int/classifications/icd10/browse/2015/en.

[5] Python Software Foundation Python Language Reference, Version 2.7. http://www.python.org/.

[6] Injury Prevention & Control: Data & Statistics (WISQARSTM): Atlanta: Centers for Disease Control and Prevention (U.S.), 5.3 Definitions for Years of Potential Life Lost. http://www.cdc.gov/injury/wisqars/fatal_help/definitions_ypll.html.

[7] National Center for Health Statistics (2014). Health, United States, 2014: With Special Feature on Adults Aged 55–64.

[8] (2014). Informe de la Salud de Puerto Rico. http://www.salud.gov.pr/Estadisticas-Registros-y-Publicaciones/EstadisticasVitales/InformedelaSaludenPUertoRico2014.pdf.

[9] R Core Team (2015). R: A Language and Environment for Statistical Computing.

[10] Dean A.G., Arner T.G., Sunki G.G., Friedman R., Lantinga M., Sangam S., Zubieta J.C., Sullivan K.M., Brendel K.A., Gao Z. (2011). Epi Info, a Database and Statistics Program for Public Health Professionals.

[11] Atlas PR. http://miguelrios.github.io/atlaspr/.

[12] SalHUD Source Code. https://github.com/humberto-ortiz/salHUD.

